# Meta-analysis of blood indices and production physiology of broiler chickens on dietary fermented cassava intervention

**DOI:** 10.1007/s11250-023-03783-1

**Published:** 2023-10-21

**Authors:** I.P. Ogbuewu, M. Mabelebele, C.A. Mbajiorgu

**Affiliations:** 1 Department of Animal Science and Technology, Federal University of Technology, P.M.B, Owerri, Imo State 1526 Nigeria; 2 Department of Agriculture and Animal Health, University of South Africa, Florida Science Campus, Private Bag X6, Florida, 1710 South Africa

**Keywords:** Poultry, Non-conventional feedstuff, Growth performance, Carcass traits, Blood characteristics

## Abstract

The effects of dietary fermented cassava on the blood constituents and production parameters of broiler chickens have been reported with variable outcomes. Therefore, this investigation aimed to explore the impacts of dietary fermented cassava on growth traits, blood constituents, visceral organ, and carcass characteristics of broiler chickens. Four databases were searched for studies that assessed responses of broiler chickens dietary fermented cassava. Eleven articles were used for the investigation, and data generated were analysed using OpenMEE software. A random effects model was used, and effect sizes were presented as standardised mean difference (SMD) at a 95 % confidence interval (CI). Sources of heterogeneity were evaluated using the following modifiers: broiler strain used, cassava form, feeding duration, type of microbes used for the fermentation, and inclusion level of cassava. Results indicate that fermented cassava-based diets increased feed intake (SMD = 0.38; 95 % CI: 0.11, 0.65; *P* = 0.006), feed conversion ratio (SMD = 1.26; 95 % CI: 0.91, 1.61; *P* < 0.001), white blood cells (SMD = 1.26; 95 % CI: 0.54, 1.98; *P* < 0.001), total serum protein (SMD = 1.23; 95 % CI: 0.41, 2.05; *P* = 0.003), serum cholesterol (SMD = 0.43; 95 % CI: 0.01, 0.85; *P* = 0.050), serum creatinine (SMD = 2.53; 95 % CI: 0.53, 4.53; *P* = 0.013), and serum uric acid (SMD = 4.33; 95 % CI: 6.25, 2.41; *P* < 0.001), but lowered average daily gain and carcass yield, taking heterogeneity into account. Results reveal that studied modifiers were responsible for the inconsistent results among authors. In conclusion, dietary fermented cassava negatively influenced carcass yield, growth performance, and aspects of blood indices of broiler chickens, but did not affect abdominal fat content, visceral organ weights, and cut-part weights. However, more innovative research is needed to improve the feeding quality of cassava using other biotechnological tools in order to maximise its potential as an energy source in broiler chickens.

## Introduction

The potential contribution of poultry in improving household food security and nutrition in developing countries has been highlighted (Birhanu et al. 2023). However, the contribution of poultry to enhancing food security is limited by the high cost of conventional energy feedstuffs such as maize. Maize constitutes over 50% of the poultry diets (Chukwukaelo et al. 2018; Ogbuewu and Mbajiorgu 2022). The use of maize for food, animal feed, and brewing industry, among others, creates stiff competition among these end users, which in turn pushes the price of maize beyond the limits required for cost-effective and sustainable production of poultry feeds in developing countries. Thus, this has necessitated the use of cassava (Manihot species) as alternative energy sources for poultry (Aladi 2016; Chukwukaelo et al. 2018; Ogbuewu and Mbajiorgu 2022).

Cassava is non-seasonal and drought-resistant plant and yields about 5 to 10 times more calories per unit area of land than maize (Ogbuewu and Mbajiorgu 2023a). It belongs to the kingdom Plantae and the family Euphorbiaceae. Cassava is a woody plant with erect stems, and the roots are usually cylindrical. It is categorised as either a bitter or sweet variety. According to Ogbuewu and Mbajiorgu (2023a), cassava root, cassava starch residue, peels, pulps, chips, and root sievate were classified as sources of energy in chicken feed and are known as co-energy products, whereas cassava leaf is used as a source of dietary protein. The use of cassava as a co-energy product in animal nutrition has been advocated primarily because it is readily available and less expensive than maize. The root and peel meals of cassava are high in energy, but their use as a source of energy in chicken feed is limited by the dustiness of its dried meal, high concentrations of cyanogenic glycosides (i.e. linamarin and lotaustralin) which yield hydrocyanic acid (HCN) on hydrolysis (Aladi 2016; Abouelezz et al. 2018; Ogbuewu and Mbajiorgu 2023b). Cassava is also low in protein (≤ 3.0%) with poor essential amino acid compositions, especially cysteine and methionine. The fibrous nature of cassava by-products may limit their digestion and uptake as the chicken gut was not designed to handle fibrous diets. The application of biotechnology to enhance protein biomass and fibre digestion and reduce anti-nutrient agents in cassava has been advocated (Aladi 2016; Chukwukaelo et al. 2018; Ogbuewu and Mbajiorgu 2023a). Fermented cassava is rich in nutrients and unknown growth factors produced by microbes during the fermentation process (Chukwukaelo et al. 2018). As a result, fermented cassava can be an excellent source of energy for broiler chickens. A recent review by Ogbuewu and Mbajiorgu (2023a) revealed variable performance results in avian species offered fermented cassava.

The varying results in broilers offered fermented cassava could be ascribed to differences in strains of broilers used, cassava variety, type of microorganisms used for the fermentation, diet composition, and quantity of fermented cassava included in the ration shown to affect chicken performance (Ogbuewu and Mbajiorgu 2022). The use of meta-analysis (an advanced statistical method that combined multiple studies on the same topic) to resolve studies with conflicting findings has been reported in the literature (Ogbuewu and Mbajiorgu 2023b; Rodrigues et al. 2023). Therefore, this study aimed to use meta-analysis to explore the impact of fermented cassava on growth performance, visceral organ weight, carcass traits, and blood indices of broiler chickens.

## Materials and methods

### Literature search

**Fig. 1:**
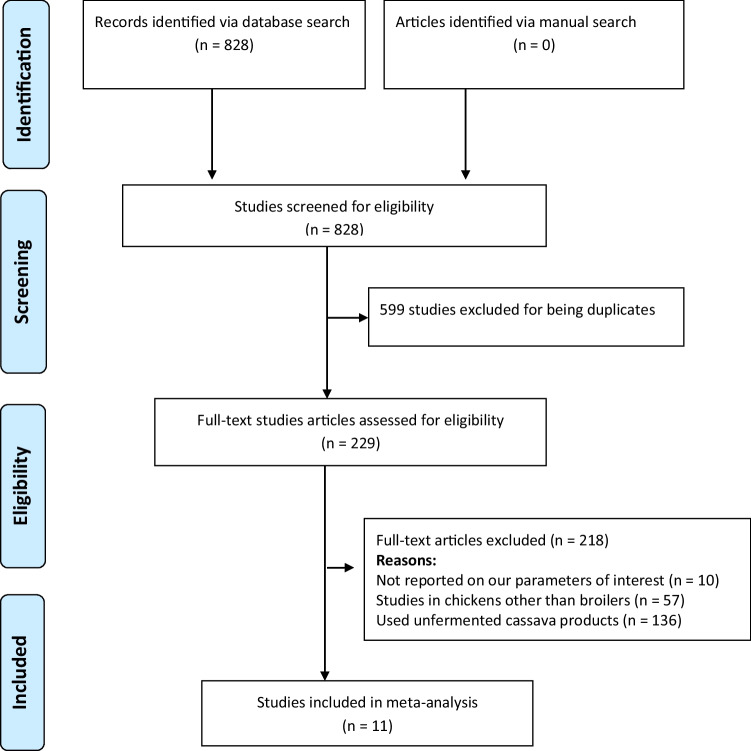
Article selection flow chart

The research question for the present study was formulated using the PICO format, where population (P): broiler chickens; intervention (I): dietary fermented cassava; comparison (C): ration with and without fermented cassava; and the outcomes (O): performance indicators, e.g. growth performance, visceral organ weight, abdominal fat, carcass traits, and blood characteristics. PubMed, Scopus, Google Scholar and Web of Science databases were searched for controlled studies that evaluated the effect of dietary fermented cassava on performance indicators of broiler chickens using PRISMA guidelines as shown in Fig. 1. The search was performed using a blend of Boolean logic (AND/OR) and the following keywords: fermented cassava, broiler chickens, growth performance, visceral organ, carcass weight, and blood indices. The search strategies for the four online databases are shown in Table 1. Studies that appeared in more than one database were considered as duplicates and duplicate articles were not used for the analysis.

**Table 1:** Search strategies for PubMed, Scopus, Google Scholar and Web of Science databases

Database	Search terms	Number of studies identified
PubMed	(“fermented cassava” OR “fermented cassava products”) AND (“broilers” OR “broiler chickens”) AND (“growth performance” OR “blood” OR “carcass” OR “cut part weight” OR “visceral organs”)	205
Scopus	(“fermented cassava” OR “fermented cassava products”) AND (“broilers” OR “broiler chickens”) AND (“growth performance” OR “blood” OR “carcass” OR “cut part weight” OR “visceral organs”)	187
Google Scholar	(“fermented cassava” OR “fermented cassava products”) AND (“broilers” OR “broiler chickens”) AND (“growth performance” OR “blood” OR “carcass” OR “cut part weight” OR “visceral organs”)	356
Web of Science	(“fermented cassava” OR “fermented cassava products”) AND (“broilers” OR “broiler chickens”) AND (“growth performance” OR “blood” OR “carcass” OR “cut part weight” OR “visceral organs”)	80

### Eligibility conditions

The following inclusion criteria were used: (1) controlled trials that investigated the influence of fermented cassava rations on measured outcomes in broiler chickens and (2) studies that measured at least one of the following parameters: growth performance, blood characteristics, visceral organ weight, abdominal fat weight, and carcass traits. Exclusion criteria included (1) review articles and trials not performed in broiler chickens were removed; (2) trials that used non-fermented cassava products were excluded; (3) trials not reported on our parameters of interest; and (4) non-controlled studies were not used for the study. Eight hundred and twenty eight (828) studies were collected from methodical searches performed on the four databases of which 599 duplicates were removed. Two hundred and eleven (211) trials were also excluded after screening the abstracts and abstracts, leaving 18 full-text studies. After reviewing the full-text articles, additional seven publications were removed for not satisfying the eligibility conditions. Eleven full-text articles were used for the meta-analysis as illustrated in Fig. 1.

### Data extraction

Data were extracted on study identification (surname of the first author, publication year); study country (Thailand, Indonesia, Nigeria, and Ghana); continent (Asia and Africa), growth indices: feed intake (FI), feed conversion ratio (FCR), and average daily gain (ADG); visceral organs (liver, gizzard, and heart); abdominal fat; carcass traits (yield, breast, drumstick, and thigh); haematology [packed cell volume (PCV), haemoglobin (Hb), red blood cells (RBC), white blood cells (WBC)]; and blood chemistry: total protein, cholesterol, low-density lipoprotein (LDL), high-density lipoprotein (HDL), triglycerides, creatinine, uric acid, aspartate aminotransferase (ALT), and alanine aminotransferase (ALT). Data were also collected on the following modifiers: cassava form (chip, pulp, stump, whole root, peels, starch residue, and roots), broiler strains (Arbor Acres, Lohmann, Ross, Cobb, Anak, and Hubbard), feeding duration (1–63 days), inclusion level (0–60%), and microbes used for the fermentation (Yeast, *Acremonium charticola*, *Bacillus subtilis*, *Aspergillus niger*, *A. oryzae*, *Lactobacillus delbrueckii*, *L. coryniformis*, and *A. fumigatus*). Authors whose studies were used for the analysis did not state the variety of cassava (sweet versus bitter) they used, and as a result, its effect on response variables was not determined in the present study.

### Statistical analysis

The analyses were done using the OpenMEE software (Wallace et al. 2016). The effect of fermented cassava intervention on broiler chicken performance was assessed using a random effects model following the method of DerSimonian and Laird (2015). The impact of fermented cassava on measured outcomes was assessed using SMD (95 % CI). The *Q*-statistic and *I*^2^-statistic were used to assess heterogeneity (Higgins and Thompson 2002). Publication bias was determined by the Rosenberg fail-safe (Nfs) number. Blood characteristics, carcass traits, and visceral organ weights were not subjected to publication bias and meta-regression analysis since the number of number of experiments needed to carry-out these analyses was less than 10 (Borenstein et al. 2010). All the analyses were considered significant at 5% probability level. The mean pooled result is deemed significant when the lower and upper CIs excluded zero. The points to the left of the line of no effect (SMD = 0) connote a reduction in the values of FI, FCR, and ADG, while the points to the right depict an increase in the values of FI, FCR, and ADG. Pooled results were deemed robust in the presence of publication bias if Nfs > 5N_study_ + 10), where *n* is the number of studies (Jennions et al. 2013).

## Results

The systematic search executed on the four databases yielded 828 prospective studies of which 11 met the predetermined inclusion criteria. Two thousand one hundred and six (2106) broiler chickens comprising 1738 chickens for the experimental group and 368 chickens for the control group were used for the analysis Microbes used for the cassava fermentation were yeast, *Acremonium charticola*, *Bacillus subtilis*, *Aspergillus niger*, *A. fumigatus*, *A. oryzae*, *Lactobacillus delbrueckii*, and *L. coryniformis*. Fermented cassava was offered to broiler chickens as dried meals. Published articles used for the investigation were performed in four study countries (Thailand, Indonesia, Nigeria, and Ghana) drawn from two continents (Africa and Asia). Arbor Acres, Lohmann, Ross, Cobb, Anak, and Hubbard broiler strains were used for the meta-analysis. Broiler chickens used for the analysis were fed fermented cassava at an inclusion level of 0 to 60% and aged from 1 to 63 days. A detailed characteristics of published articles included in this study was described in Table 2. The earliest study included in the analysis was conducted in 1977, and the latest was performed in 2022. The studies used for analysis spanned 45 years.

**Table 2:** Description of studies used for the analysis

Author	Country	Continent	C^+^	Modifiers					No. of chickens	Parameters measured
				Cassava form	Inclusion level (%)	Broiler strains	RD (days)	Microbes used for the study		
Pido et al. (1979)	Nigeria	Africa	4	Whole root	0, 25, 37.5, 50	Cobb	7–63	*Aspergillus* species	160	FI, FCR, ADG, carcass yield, liver, heart, gizzard, abdominal fat
Osei and Duodu (1988)	Ghana	Africa	4	Peels	0, 5, 10, 15	Cobb	1–42	*Aspergillus* *oryzae*	480	FI, FCR, ADG,
Ali-Mursyid et al. (2010)	Indonesia	Asia	4	Peeled root	0, 10, 20, 30	Hubbard	1–35	*Aspergillus* *niger*	120	FI, FCR, ADG
Aro et al. (2012)	Nigeria	Africa	4	Peels/starch residues	0, 20, 40, 60	Ross	1–21	Lactobacillus *delbrueckii*, *Lactobacillus coryniformis*, *Aspergillus fumigatus*	200	FI, FCR, ADG, PCV, RBC, Hb, cholesterol, total protein, ALT, AST,
Khempaka et al. (2014)	Thailand	Asia	6	Pulp	0, 4, 8, 12, 16, 20	Arbor Acres	1–42	*Aspergillus* *oryzae*	270	FI, FCR, carcass yield, breast, thigh, drumstick, abdominal fat, heart, liver, gizzard
Sugiharto et al. (2016)	Indonesia	Asia	4	Peeled root	0, 2.5, 5, 10	Lohmann	8–35	*Aspergillus* niger	160	FI, FCR, ADG, RBC, WBC, AST, ALT, triglycerides, total protein, liver, carcass yield, abdominal fat
Ojewola et al. (2016)	Nigeria	Africa	11	Peeled root	0, 5.68, 11.72, 17.58, 23.44, 29.3, 35.16, 41.02, 46.88, 52.74, 58.6	Anak	1–56	*Aspergillus* species	220	FI, FCR, ADG
Sugiharto et al. (2017)	Indonesia	Asia	2	Pulp	0, 16	Lohmann	11–35	*Acremonium charticola*	56	ADG, FCR, heart, gizzard, carcass yield, breast, thigh, drumstick, abdominal fat
Sugiharto et al. (2019)	Indonesia	Asia	4	Pulp	0, 10, 15, 20	Lohmann	22–38	*Acremonium charticola*, *Bacillus subtilis*	200	FI, FCR, ADG, heart, liver, gizzard, abdominal fat, Hb, PCV, RBC, WBC, ALT, AST, uric acid, creatinine, triglycerides, cholesterol, LDL, HDL, total protein, breast, thigh, drumstick, carcass yield
Animashahun et al. (2022)	Nigeria	Africa	4	Stump	0, 13, 26, 39	Ross	1–56	*Aspergillus niger*	120	Carcass yield, breast, thigh, drumstick, gizzard, abdominal fat
Thip-uten et al. (2022)	Thailand	Asia	4	Chip	0, 10, 20, 30	Arbor Acres	11–42	Yeast	120	FI, FCR, ADG, creatinine, uric acid, cholesterol, triglycerides, HDL, LDL, AST, ALT, breast, thigh, drumstick, liver, gizzard, abdominal fat

*C*^*+*^ number of comparisons, *FD* feeding duration, *FI* feed intake, *FCR* feed conversion ratio, *ADG* average daily gain, *RBC* red blood cell, *WBC* white blood cell, *Hb* haemoglobin, *PCV* packed cell volume, *LDL* low-density lipoprotein cholesterol, *HDL* high-density lipoprotein cholesterol, *AST* aspartate aminotransferase, *ALT* alanine aminotransferase

### Growth performance

#### Feed intake (FI)

**Fig. 2:**
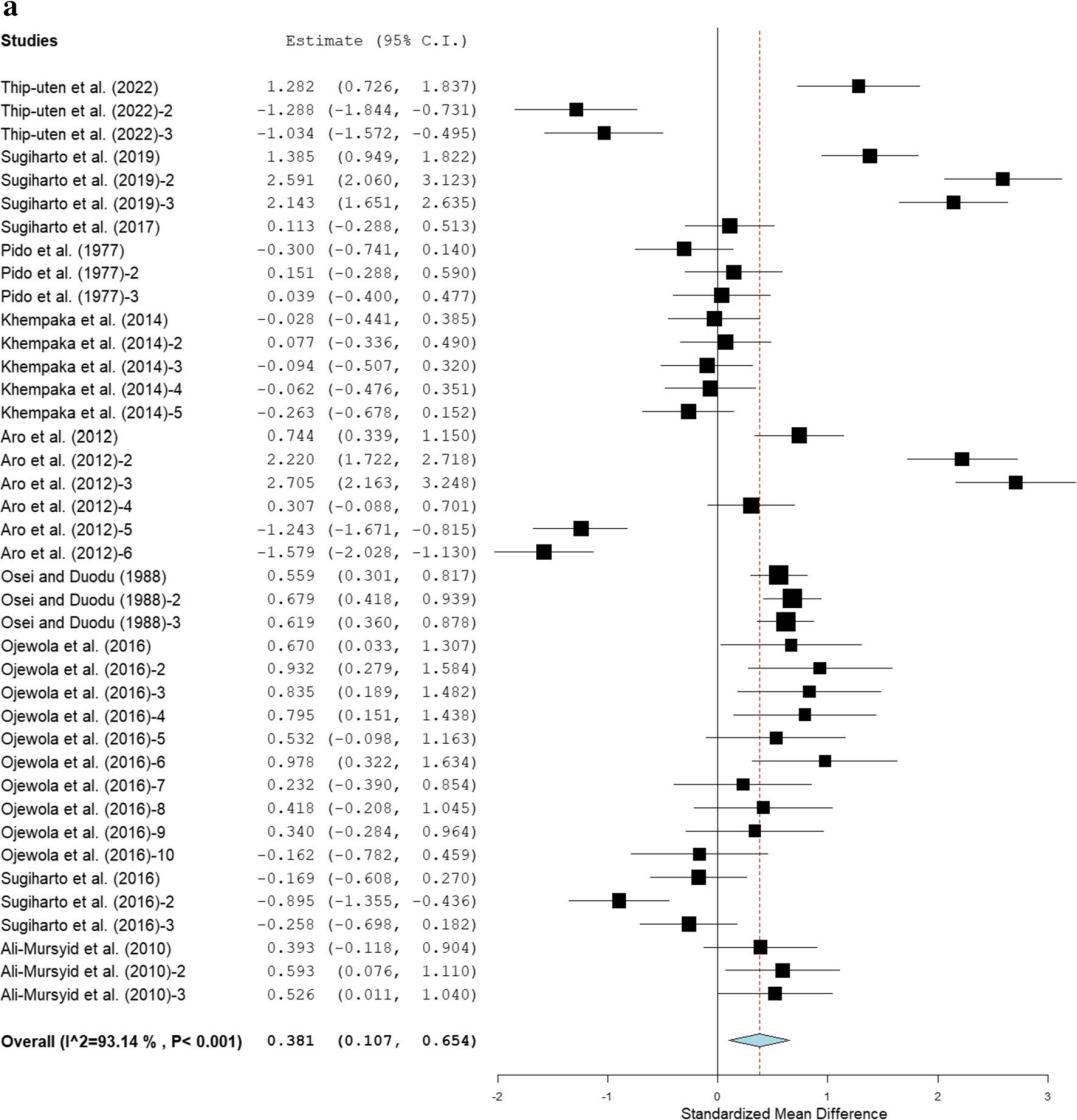
Forest plot of FI of broiler chickens fed fermented cassava. *CI* confidence interval; *FI* feed conversion ratio; *I*^*2*^ Inconsistency index. The solid vertical line depicts a mean difference of zero (0) or no effect. Points to the left of the no effect line (zero) depict a decrease in FI, and opposite depicts a decrease in F1. Individual square in the plot represents the mean effect size for each experiment, while the upper and lower 95% CI for the effect size are the line that joined the squares. The dotted line with the diamond at the base showing the 95% CI depicts the pooled estimation. *I*^*2*^ = inconsistency index is a measure of variance above chance among articles utilised in the analysis. Pooled estimation is considered significant when the line of no effect did not touch the diamond at the bottom of the forest plot (Koricheva et al. 2013).

Figure 2 presents the FI values of broiler chickens that received fermented cassava diets. Fermented cassava increased FI when compared to the control (SMD = 0.38; 95 % CI: 0.11, 0.65; *P* = 0.006; *I*^*2*^ = 93.14 %). Subgroup analysis by broiler strains as illustrated in Table 3 showed that Anak, Cobb, and Hubbard strains had significantly increased FI when compared to the control, whereas Ross, Lohmann, and Arbor Acres strains had similar FI values with the control. Broiler chickens on dietary fermented cassava peel and root meal recorded higher significantly FI than the control. On the other hand, fermented whole cassava roots, chips, starch residues, and pulps did not affect FI in broiler chickens. Broiler chickens fed cassava fermented with a mixture of *A. charticola* and *Bacillus subtilis* had increased FI. In contrast, broiler chickens fed cassava co-energy products fermented with yeast (*Saccharomyces cerevisiae*), *A. oryzae*, and mixtures of *L. delbrueckii*, *L. coryniformis*, and *A. fumigatus* had similar FI values with the control group. Higher FI (SMD = 0.38; 95 % CI: 0.15, 0.61; *P* = 0.002) was recorded in broiler chickens offered cassava fermented with microbes for > 42 days, but those offered fermented cassava diets for 1–21 days and 1–42 days had comparable FI with the control. Broiler chickens that received fermented cassava at 11–20 % recorded higher FI. Heterogeneity was observed across trials that explore the impact of fermented cassava on FI (*P* < 0.001, *I*^*2*^ = 93.14 %; Fig. 2). To explore the origin of heterogeneity in the present study, the following modifiers were considered: broiler strains, inclusion level, cassava form, microbes used for fermentation, and feeding duration (Table 4). There is a significant relationship between modifiers (cassava form and feeding duration) and FI in broiler chickens and modifiers accounted for 46 % (24 % and 22 %, respectively) of the sources of variations.

**Table 3:** Effect of fermented cassava on FI of broiler chickens

Variables	Subgroup	C+	SMD (95% CI)		Heterogeneity	
			Random effects	*P-value*	*I*^*2*^	*P-value*
Broiler strains	Arbor acres	8	−0.17 (−0.62, 0.28)	0.451	87.02	< 0.001
	Lohmann	7	0.70 (−0.23, 1.63)	0.142	96.61	< 0.001
	Cobb	6	0.33 (0.06, 0.61)	0.019	76.84	< 0.001
	Ross	6	0.52 (−0.78, 1.82)	0.433	98.03	< 0.001
	Anak	10	0.55 (0.32, 0.77)	< 0.001	18.52	0.273
	Hubbard	3	0.50 (0.21, 0.80)	< 0.001	0.00	0.861
Cassava form	Chip	3	−0.35 (−1.94, 1.25)	0.669	96.02	< 0.001
	Pulp	9	0.64 (−0.03, 1.29)	0.051	95.00	< 0.001
	Whole root	3	−0.04 (−0.30, 0.23)	0.789	8.83	0.335
	Peels	6	1.22 (0.66, 1.77)	< 0.001	94.03	< 0.001
	Starch residue	3	−0.84 (−2.00, 0.34)	0.162	95.83	< 0.001
	Peeled root	16	0.33 (0.06, 0.60)	0.016	72.66	< 0.001
Microbes	Yeast	3	-0.35 (−1.94, 1.25)	0.669	96.02	< 0.001
	A^+^	3	2.03 (1.32, 2.73)	< 0.001	84.14	< 0.001
	A. oryzae	5	−0.07 (−0.26, 0.11)	0.434	0.00	0.849
	A^++^	6	0.52 (−0.78, 1.82)	0.433	98.03	< 0.001
	Fungus	3	−0.44 (−0.88, 0.01)	0.053	66.00	0.053
Feeding duration (days)	1–21	6	0.52 (−0.78, 1.82)	0.433	98.03	< 0.001
	1–42	21	0.33 (−0.01, 0.67)	0.059	93.06	< 0.001
	>42	13	0.38 (0.15, 0.61)	0.002	53.16	0.012
Inclusion level (%)	1–10	11	0.33 (−0.03, 0.69)	0.073	88.59	< 0.001
	11–20	14	0.54 (0.10, 0.98)	0.015	92.76	< 0.001
	21–30	4	−0.02 (−0.80, 0.76)	0.958	87.25	< 0.001
	31–40	4	0.52 (−0.98, 2.02)	0.497	97.31	< 0.001
	41–50	3	0.18 (−0.13, 0.49)	0.255	0.00	0.612
	>50	4	0.32 (−1.59, 2.23)	0.739	97.91	< 0.001

*A*^*+*^, *A. charticola* + *Bacillus subtilis*; *A*^*++*^, *L. delbrueckii* + *L. coryniformis* + *A. fumigatus*; *SMD*, standardised mean difference; *C*^*+*^, number of comparison; *CI*, confidence interval; *P*, probability; *I*^*2*^, inconsistency index

**Table 4:** Meta-regression of the impact of fermented cassava on growth traits of broiler chickens

Variables	Moderators	Intercept	Q_M_	DF	*P*-value	*R*^2^ (%)
FI (g/bird/day)	Strains	−0.17	3.87	5	0.569	0.00
	Feeding duration (day)	−0.35	18	8	0.021	21.85
	Microbes	−0.35	9.61	5	0.087	19.33
	Inclusion level (%)	0.70	8.74	28	1.000	0.00
FCR	Form	1.24	9.18	5	0.102	8.18
	Strains	0.62	99.4	5	< 0.001	75.03
	Feeding duration (day)	1.23	106	8	< 0.001	75.90
	Microbes	1.24	14.3	5	0.014	33.11
	Inclusion level (%)	−0.16	80.1	28	6.51E-07	60.00
ADG (g/bird/day)	Form	−3.10	19.9	5	0.0013	31.35
	Strains	−3.00	88.2	5	< 0.001	74.32
	Feeding duration (day)	−2.95	124	8	< 0.001	81.24
	Microbes	−3.02	34.2	4	6.66E-07	70.05
	Inclusion level (%)	0.36	48.4	25	0.003	41.89

*FI* feed intake, *FCR* feed conversion ratio, *ADG* average daily gain, *DF* degree of freedom, *Q*_*M*_ coefficient of moderator, *P* probability, *R*^2^ amount of heterogeneity explained by moderators, *CI* confidence interval

#### Feed conversion ratio (FCR)

**Fig. 3:**
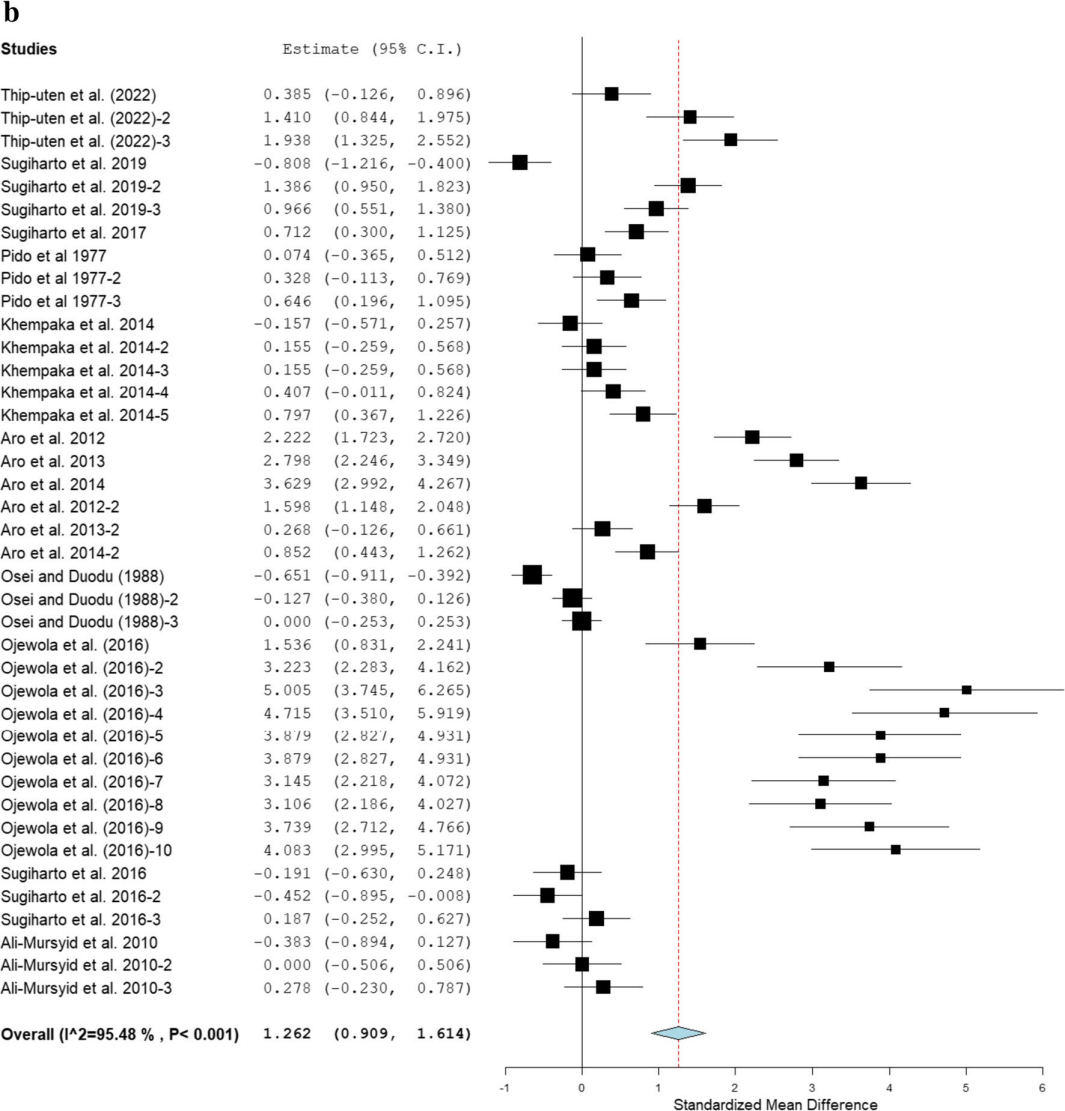
Forest plot of the effect of fermented cassava on FCR of broiler chickens. *CI* confidence interval; *FCR* feed conversion ratio; *I*^*2*^ inconsistency index. The solid vertical line depicts a mean difference of zero (0) or no effect. Points to the left of the no effect line (zero) depict a decrease in FCR, and opposite depicts a decrease in FCR. Individual square in the plot represents the mean effect size for each experiment, while the upper and lower 95% CI for the effect size are the line that joined the squares. The dotted line with the diamond at the base showing the 95% CI depicts the pooled estimation. *I*^*2*^ = inconsistency index is a measure of variance above chance among articles utilised in the analysis. Pooled estimation is considered significant when the line of no effect did not touch the diamond at the bottom of the forest plot (Koricheva et al. 2013).

The pooled effect of fermented cassava on FCR in broiler chickens is shown in Fig. 3. In comparison with the controls, pooled results show that fermented cassava increased FCR (SMD = 1.26; 95% CI: 0.91, 1.61; *P* < 0.001; *I*^*2*^ = 95.48%) in broiler chickens. Table 5 showed the effects of fermented cassava on FCR of broiler chickens. Results showed that Arbor Acres, Ross, and Anak strains had significantly higher FCR than the control, but Cobb, Lohmann, and Hubbard strains had comparable FCR with the control. Broilers offered fermented cassava diets had significantly poor FCR, except for those given fermented cassava pulps that had comparable FCR with control. Broiler chickens fed cassava fermented with yeast and a mixture of *A. charticola* and *Bacillus subtilis* had poor FCR. Conversely, broilers that received diets fermented with *A. oryzae* and a mixture of *A. charticola* and *Bacillus subtilis* had similar FCR with those in the control group. Broiler chickens that received fermented cassava for a period of 1–21 days (SMD = 1.88; 95% CI: 0.93, 2.83; *P* < 0.001) and > 42 days (SMD = 2.81; 95% CI: 1.86, 3.76; *P* < 0.001) had poor FCR, but those fed fermented cassava for 1–42 days had similar FCR with control. Results indicate that inclusion level increased FCR of broiler chickens, except those given fermented cassava at 1–10% that had similar FCR with the control (SMD = −0.09; 95% CI: −0.38, 0.20; *P* = 0.52). Heterogeneity exists among the trials used for the investigation (*P* < 0.001, *I*^*2*^ = 95.48%; Fig. 3). Table 4 shows that there were relationships between FCR and modifiers (i.e. broiler strains, feeding duration, cassava form, and microbes used for fermentation).

**Table 5:** FCR of broiler chickens on dietary fermented cassava intervention

Variables	Subgroup	C+	SMD (95% CI)		Heterogeneity	
			Random effects	*P-value*	*I*^*2*^	*P-value*
Broiler strains	Arbor Acres	8	0.61 (0.18, 1.04)	0.006	85.5	< 0.001
	Lohmann	7	0.26 (−0.34, 0.85)	0.397	96.66	< 0.001
	Cobb	6	0.02 (−0.32, 0.36)	0.925	84.73	< 0.001
	Ross	6	1.88 (0.93, 2.83)	< 0.001	95.81	< 0.001
	Anak	10	3.57 (2.90, 4.25)	< 0.001	78.41	< 0.001
	Hubbard	3	−0.03 (−0.41, 0.34)	0.191	38.65	0.196
Cassava form	Chip	3	1.23 (0.32, 2.14)	0.008	87.1	< 0.001
	Pulp	9	0.40 (−0.03, 0.83)	0.067	89.42	< 0.001
	Whole root	3	0.35 (0.02, 0.67)	0.036	37.33	0.203
	Peels	6	1.28 (0.18, 2.39)	0.023	98.38	< 0.001
	Starch residue	3	0.90 (0.16, 1.64)	0.018	89.48	< 0.001
	Peeled root	16	2.16 (1.30, 3.01)	< 0.001	96.11	< 0.001
Microbes	Yeast	3	1.23 (0.32, 2.14)	0.008	87.31	< 0.001
	A^+^	3	0.51 (−0.82, 1.84)	0.449	96.68	< 0.001
	A. oryzae	5	0.27 (−0.04, 0.58)	0.088	63.21	0.028
	A^++^	6	1.88 (0.93, 2.83)	< 0.001	95.81	< 0.001
	Fungus	3	−0.15 (−0.51, 0.21)	0.416	50.91	0.130
Feeding duration (days)	1-42	21	0.27 (−0.01, 0.54)	0.055	89.75	< 0.001
	>42	13	2.81 (1.86, 3.76)	< 0.001	95.28	< 0.001
	1-21	6	1.88 (0.93, 2.83)	< 0.001	95.81	< 0.001
Inclusion level (%)	1-10	13	−0.09 (−0.38, 0.20)	0.523	82.24	< 0.001
	11-20	14	1.18 (0.71, 1.64)	< 0.001	93.09	< 0.001
	21-30	4	2.59 (0.61, 4.58)	0.010	96.6	< 0.001
	31-40	4	1.76 (0.29, 3.24)	0.019	96.73	< 0.001
	41-50	3	2.27 (0.38, 4.15)	0.018	94.68	< 0.001
	>50	4	3.04 (1.20, 4.89)	0.001	96.26	< 0.001

*A*^*+*^, *A. charticola* + *Bacillus subtilis*; *A*^*++*^, *L. delbrueckii* + *L. coryniformis* + *A. fumigatus*; *SMD*, standardised mean difference; *C*^*+*^, number of comparison; *CI*, confidence interval; *P*, probability; *I*^*2*^, inconsistency index

#### Average daily gain (ADG)

**Fig. 4:**
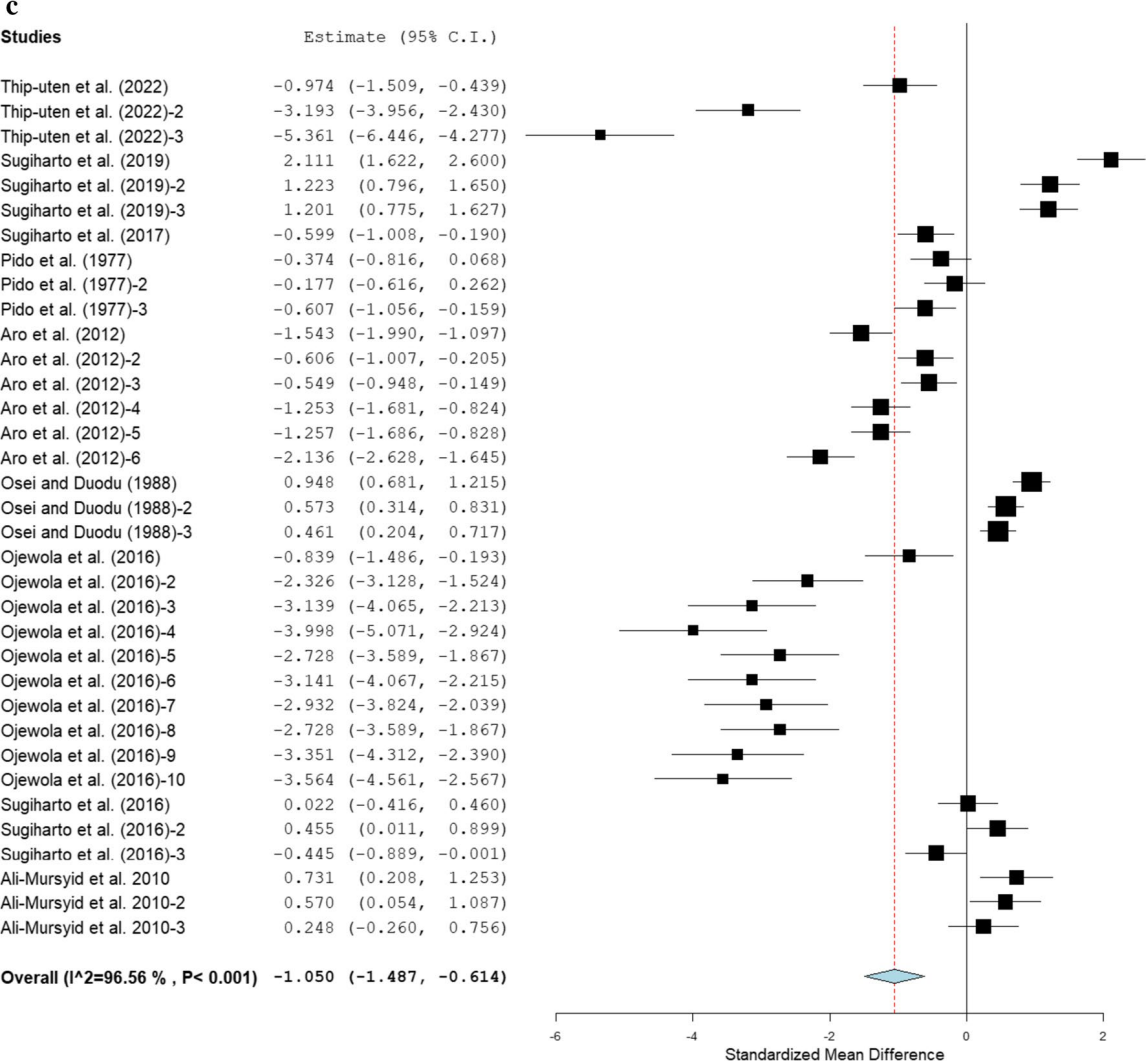
Forest plot of ADG of broiler chickens on dietary fermented cassava. *CI* confidence interval; *ADG* daily weight gain; *I*^*2*^ inconsistency index. The solid vertical line depicts a mean difference of zero (0) or no effect. Points to the left of the no effect line (zero) depict a decrease in ADG, and opposite depicts a decrease in ADG. Individual square in the plot represents the mean effect size for each experiment, while the upper and lower 95% CI for the effect size are the line that joined the squares. The dotted line with the diamond at the base showing the 95% CI depicts the pooled estimation. *I*^*2*^ = inconsistency index is a measure of variance above chance among articles utilised in the analysis. Pooled estimation is considered significant when the line of no effect did not touch the diamond at the bottom of the forest plot (Koricheva et al. 2013).

The ADG value of broilers offered fermented cassava is shown in Fig. 4. Fermented cassava-based diets reduced ADG (SMD = 1.26; 95 % CI: −1.49, −0.61; *P* < 0.001; *I*^*2*^ = 96.56 %) in broiler chickens. The influence of modifiers on ADG in broilers offered fermented cassava is described in Table 6. Fermented cassava lowers the ADG in Arbor Acres, Ross, and Anak strains, but had no effect on Cobb and Lohmann strains. In contrast, fermented cassava intervention increased ADG in Hubbard strain without heterogeneity (*P* = 0.416; *I*^*2*^ = 0 %). Fermented cassava chips, whole roots, starch residues, and peeled roots had a reduction effect on ADG. However, broiler chickens fed fermented cassava pulp meal and fermented cassava peel meal had similar ADG to the control. Conversely, broiler chickens fed cassava fermented with yeast and a mixture of *A. charticola* and *Bacillus subtilis* had poor ADG. Interestingly, broiler chickens fed cassava fermented with a blend of *A. charticola* and *Bacillus subtilis* had improved ADG. In addition, broilers given fermented cassava for 1–21 days and > 42 days experienced poor ADG. However, ADG values were not affected in birds fed test diets for the duration of 1 to 42 days. Broiler chickens that received fermented cassava diets at 21–30, 31–40, 41–50, and > 50 % had significantly reduced ADG. Results indicate that broiler chickens fed fermented cassava at 1–10 % and 11–20 % had similar ADG, putting heterogeneity into account (*P* < 0.001; *I*^*2*^ = 93.40−96.74 %; Table 6). Table 4 found significant associations between ADG and studied modifiers.

**Table 6:** ADG value of broiler chickens given fermented cassava

Variables	Subgroup	C+	SMD (95% CI)		Heterogeneity	
			Random effects	*P-value*	*I*^*2*^	*P-value*
Broiler strains	Arbor Acres	3	−3.14 (−5.54, −0.74)	0.010	96.63	< 0.001
	Lohmann	7	0.56 (−0.16, 1.28)	0.124	94.68	< 0.001
	Cobb	6	0.17 (−0.28, 0.61)	0.471	91.06	< 0.001
	Ross	6	−1.21 (−1.68, −0.75)	< 0.001	85.6	< 0.001
	Anak	10	−2.83 (−3.45, −2.22)	< 0.001	79.85	< 0.001
	Hubbard	3	0.51 (0.21, 0.81)	< 0.001	0.00	0.416
Cassava form	Chip	3	−3.14 (−5.54, −0.74)	0.010	96.63	< 0.001
	Pulp	4	0.98 (−0.14, 2.10)	0.087	96.21	< 0.001
	Whole root	3	−0.38 (−0.64, −0.13)	0.003	0.00	0.405
	Peels	6	−0.10 (−0.77, 0.56)	0.762	96.14	< 0.001
	Starch residue	3	−1.54 (−2.09, −0.99)	< 0.001	77.68	0.011
	Peeled root	16	−1.64 (−2.41, −0.88)	< 0.001	95.54	< 0.001
Microbes	Yeast	3	−3.14 (−5.54, −0.74)	0.010	96.63	< 0.001
	A^+^	3	1.50 ( 0.94, 2.06)	< 0.001	78.75	< 0.001
	A^++^	6	−1.21 (−1.68, −0.75)	< 0.001	85.60	0.028
	Fungus	3	0.01 (−0.50, 0.52)	0.967	74.71	0.019
Feeding duration (days)	1–42	16	−0.04 (−0.55, 0.46)	0.872	95.76	< 0.001
	> 42	13	−2.25 (−3.01, −1.49)	< 0.001	93.46	< 0.001
	1–21	6	−1.213 (−1.68, −0.75)	< 0.001	85.60	< 0.001
Inclusion level (%)	1–10	9	0.30 (−0.23, 0.83)	0.264	93.40	< 0.001
	11–20	11	−0.72 (−1.49, 0.06)	0.071	96.74	< 0.001
	21–30	4	−3.08 (−5.44, −0.72)	0.010	97.04	< 0.001
	31–40	4	−1.20 (−2.08, −0.33)	0.007	91.98	< 0.001
	41–50	3	−2.06 (−3.73, −0.38)	0.016	93.83	< 0.001
	> 50	4	−2.35 (−3.73, −0.96)	< 0.001	94.90	< 0.001

*A*^*+*^, *A. charticola* + *Bacillus subtilis*; *A*^*++*^, *L. delbrueckii* + *L. coryniformis* + *A. fumigatus*; *SMD*, standardised mean difference; *C*^*+*^, number of comparison; *CI*, confidence interval; *P*, probability; *I*^*2*^, inconsistency index

### Publication bias analysis

The results of publication bias analysis are presented in Table 7. The Rosenberg failsafe number were 944, 2011, and 283 for FI, FCR, and ADG, respectively. The Rosenberg failsafe numbers were higher than the threshold of 60 needed to consider the mean effect size robust.

**Table 7:** Analysis of publication bias

Outcomes	SMD	Observed significance	Target significance	Nfs number	No. of study (n)	Nfs > (5N_study_ + 10)
FI	0.35	< .0001	0.05	944	10	60
FCR	0.52	< .0001	0.05	2011	10	60
ADG	−0.38	< .0001	0.05	283	10	60

*SMD* standardised mean difference, *FI* feed intake, *FCR* feed conversion ratio, *ADG* average daily gain, *n* number of study, *Nfs* fail-safe number.

### Carcass traits and visceral organ weights

The results of carcass traits and visceral organ weights of broilers offered fermented cassava-based diets are given in Table 8. Broilers fed fermented cassava diets had reduced carcass yield. In contrast, results showed no treatment effect on breast, thigh, and drumstick weights in comparison with the control. Similarly, there were no treatment effect on liver, heart, and gizzard weight in broiler chickens. Abdominal fat weight was not significantly different from the control.

**Table 8:** Carcass traits and visceral organ weight of broiler chickens fed fermented cassava

Variables	C+	SMD (95% CI)		Heterogeneity		
		Random effects	*P-value*	*X*^2^ (Q)	*I*^*2*^ (%)	*P-value*
Carcass yield	18	−0.21 (−0.37, −0.04)	0.017	45.67	63	< 0.001
Breast weight	15	−0.31 (−0.62, 0.01)	0.054	101.12	86	< 0.001
Drumstick weight	15	0.07 (−0.12, 0.26)	0.458	36.60	62	< 0.001
Thigh weight	15	0.31(−0.02, 0.65)	0.067	115.97	88	< 0.001
Liver	21	0.31 (−0.06, 0.69)	0.103	279.15	93	< 0.001
Heart	15	0.12 (−0.27, 0.51)	0.551	162.67	91	< 0.001
Gizzard	18	−0.02 (−0.46, 0.41)	0.922	267.13	94	< 0.001
Abdominal fat	21	0.19 (−0.93,1.30)	0.742	1489.46	99	< 0.001

*SMD* standardised mean difference, *C*^*+*^ number of comparison, *REM* random effects model, *CI* confidence interval, *P* probability, *I*^*2*^, inconsistency index

### Blood characteristics

Table 9 shows the results of blood constituents of broiler chickens offered fermented cassava-based diets. There was significantly higher WBC (SMD = 1.26; 95% CI: 0.54, 1.98; *P* < 0.001), total serum protein (SMD = 1.23; 95% CI: 0.41, 2.05 ; *P* = 0.003), serum cholesterol (SMD = 0.43; 95% CI: 0.01, 0.85; *P* = 0.050), serum creatinine (SMD = 2.53; 95% CI: 0.53, 4.53; *P* = 0.013), and serum uric acid (SMD = 4.33; 95% CI: 6.25, 2.41; *P* < 0.001) in broiler chickens fed fermented cassava than the controls. No treatment effect was noticed on Hb, PCV, RBC, serum triglycerides, LDL, and HDL. Broiler chickens given test diets had comparable serum ALT and AST with the controls.

**Table 9:** Effect of fermented cassava on blood characteristics of broiler chickens

Variables	C+	SMD (95% CI)		Heterogeneity		
		Random effects	*P-value*	*X*^2^ (Q)	*I*^*2*^ (%)	*P-value*
Haemoglobin	9	0.04 (−0.28, 0.37)	0.800	42.99	81	< 0.001
Packed cell volume	9	−0.05 (−0.36, 0.25)	0.729	48.34	83	< 0.001
Red blood cell	12	−0.23 (−0.61, 0.15)	0.244	111.74	90	< 0.001
White blood cell	6	1.26 (0.54, 1.98)	< 0.001	72.85	93	< 0.001
Total protein	11	1.23 (0.41, 2.05)	0.003	420.03	97	< 0.001
Triglycerides	9	−0.73 (−2.84, 1.39)	0.501	755.14	99	< 0.001
Cholesterol	12	0.43 (0.01, 0.85)	0.050	128.21	91	< 0.001
LDL	9	−0.15 (−2.33, 2.03)	0.894	820.52	99	< 0.001
HDL	6	1.33 (−0.40, 3.06)	0.132	297.71	98	< 0.001
Creatinine	6	2.53 (0.53, 4.53)	0.013	304.62	98	< 0.001
Uric acid	6	4.33 (6.25, 2.41)	< 0.001	243.90	98	< 0.001
ALT	15	−0.40 (−1.42, 0.62)	0.441	656.12	98	< 0.001
AST	15	0.61 (−0.29, 1.50)	0.183	684.21	98	< 0.001

*SMD* standardised mean difference, *C+* number of comparison, *REM* random effects model, *CI* confidence interval, *P* probability, *I*^*2*^ inconsistency index, *LDL* low-density lipoprotein, *HDL* high-density lipoprotein, *AST* aspartate aminotransferase, *ALT* alanine aminotransferase.

## Discussion

The results of this study suggest that broilers given fermented cassava had higher FI and poor FCR and ADG in comparison with the controls. The poor ADG observed in broiler chickens fed test diets suggest that diets were not well utilised by the experimental broilers. There is evidence that fermented feed is unpalatable to chickens due to high levels of biogenic amines and organic acids, leading to a reduction in feed intake (Niba et al. 2009). This was not the case in the current meta-analysis as feed intake in broilers given fermented cassava-based diets was not reduced. Higher dietary fibre intake in monogastric animals dilutes nutrient density and stimulates the flow of digesta, which in turn increases feed intake (Hetland et al. 2004). The poor growth in broilers that received fermented cassava agrees with the works of other researchers (Aladi 2016; Abouelezz et al. 2018) that cassava is low in crude protein (≤ 3.0%) with a limited amino acid profile, especially methionine that is required for optimal performance (Rehman et al. 2019). On the same hand, the observed poor growth traits in broilers fed fermented cassava may be linked to high levels of some fermentation metabolites (e.g. biogenic amine, organic acids) which may impair digestion and nutrient utilisation leading to a decrease in ADG (Omede et al. 2017).

The finding of the study showed that broiler strains are sources of heterogeneity in trials that explored the impact of fermented cassava in broiler chickens. This finding agrees with the results of Rondelli et al. (2003) that chicken genetics affects growth indices in chickens. The results of this study show that Hubbard strain fed fermented cassava-based diets had improved performance data, presumably due to their enhanced ability to utilise fermented cassava diets. More studies are therefore needed to determine the variable (s) responsible for improved weight gain in Hubbard broiler strain offered fermented cassava-based diets. The comparable ADG in Lohmann and Cobb strains when compared with the controls indicates that fermented cassava had no negative effect on broiler performance. In converse, the reduced ADG in Arbor Acres, Anak, and Ross strains suggest the low ability of these strains to utilise fermented cassava-based diets. This observation suggests that the potential to digest and utilise fermented cassava-based diets differed among broiler strains. The part of cassava used for feed formulation has been demonstrated to influence chicken productivity (Ogbuewu and Mbajiorgu 2022). The fact the broiler chickens fed fermented cassava pulp meal had numerically higher ADG than the control implies that the ability of broilers to utilise different cassava forms in their diet varies as confirmed by Ogbuewu and Mbajiorgu (2022) in broiler chickens fed differently processed cassava co-energy and co-protein products.

In recent years, it has become a common practice to use fermenting microbes to increase the nutritional quality of non-conventional feed resources for use in poultry feeding (Chukwukaelo et al. 2018; Gunun et al. 2023). The improvement in ADG in broilers offered cassava co-energy products fermented with a blend of *A. charticola* and *Bacillus subtilis* is most likely due to the ability of these microbes to hydrolyse the fibre and starch structure of cassava into short-chain polysaccharides during the fermentation process and thus improves the capability of endogenous digestive enzymes in chicken to act on the substrate. This would lead to an improvement in nutrient utilisation in broiler chickens. These findings agree with an earlier study in which feeding cassava pulp fermented with *Aspergillus oryzae* to broiler chickens improved nutrient utilisation (Khempaka et al. 2014). In contrast, the poor ADG in broiler chickens fed cassava fermented with yeast, and a blend of *L. delbrueckii*, *L. coryniformis*, and *A. fumigatus* in this study suggests the low ability of these microbes to improve the nutritional quality of cassava during the fermentation process. The ADG, FI, and FCR values of broiler chickens fed cassava fermented with *A. oryzae* and *A. niger* were not evaluated in the present study due to insufficient data. Therefore, more research is needed in this area.

Results indicate that feeding duration is a significant predictor of FI, FCR, and ADG in broilers given fermented cassava and caused most of the variations in the measured outcomes. The current study found that broilers fed fermented cassava-based diets for more than 42 days had significantly increased FI, but this did not translate into increased ADG. The same pattern was also observed in broiler chickens that received fermented cassava diets for 1 to 21 days. The non-significant difference in ADG found in this analysis for birds fed fermented cassava for 1 to 42 days suggests that fermented cassava was utilised by the experimental broilers. Meta-regression suggests that inclusion level is a predictor of treatment effect on FCR and ADG in broiler chickens. Broilers fed diets containing 1–10 % and 11–20 % fermented cassava had comparable FCR and ADG to controls, indicating that these inclusion levels are well tolerated. On the other hand, broilers that received fermented cassava at 21–30, 31–40, 41–50, and > 50 % had significantly poor ADG, as confirmed by Ogbuewu and Mbajiorgu (2022) that broilers fed moderate to high levels of processed cassava diets. This could be due to anti-nutritional factors (Omede et al. 2017; Ogbuewu and Mbajiorgu 2023a) contained present in fermented cassava-based diets, exceeding the tolerance level of the broiler chickens.

Evidence exists that cassava co-energy products are low in cysteine and methionine (Omede et al. 2017; Ogbuewu and Mbajiorgu 2023a), which are vital for muscle protein synthesis (Akter et al. 2020). The reduced carcass yield in broilers offered fermented cassava could be attributed to fermented cassava to dilute the amount of methionine in the diet. In contrast, fermented cassava did not affect the weights of the breast, drumstick, and thigh, as well as the weights of the liver, gizzard, and heart, implying that fermented cassava-based diet supported cut part yield and organ development in broiler chickens. Aladi et al. (2017) found that fermented cassava root meal did not affect abdominal fat weight in broiler birds. On the same hand, fermented cassava-based ration had no negative effect on abdominal fat content in broiler chickens, implying the low fat-forming potential of the test diets.

Blood constituents are employed in feeding studies to assess the nutritional quality of non-conventional feedstuffs (Ogbuewu et al. 2015). The present study shows that fermented cassava influenced aspects of blood values of broiler chickens. The observed significantly higher WBC suggests that the test diet may contain metabolites, which may stimulate the production of this blood component which may in turn enhance broiler chicken productivity. Total serum protein, which is a measure of the amounts of albumin and globulin in the blood, was higher in birds on fermented cassava than the control. The elevated total serum protein levels could be ascribed to an increase in the proportion of globulin which is typically high in broiler chickens fed fermented diets (Zhu et al. 2023). The significantly higher serum uric acid in birds offered fermented cassava is a pointer that proteins contained in the test diet are of poor quality and result in poor FCR and ADG. On the same hand, the higher serum creatinine levels in broiler chickens fed fermented cassava diets imply an increased breakdown of endogenous protein for energy production. The observed increase in serum creatinine levels indicates poor utilisation of nutrients contained in fermented cassava diets, resulting in poor ADG.

The significantly higher serum cholesterol levels in broilers fed fermented cassava diets compared to the controls indicates the ability of test diets to support serum cholesterol formation. Aspartate aminotransferase and ALT are enzymes found in liver cells that leak into the blood when the cells are damaged (Enemor et al. 2005). However, the comparable serum ALT and AST values in broilers offered test diets when compared to the controls ruled out the possibility of liver damage.

Publication bias undermines the reliability of meta-analysis results as journal editors prefer to publish results with positive findings over negative outcomes. Jennions et al. (2013) found that the results of pool analyses can be considered robust even in the presence of publication bias if fail-safe number is greater than (5N_study_ + 10). As a result, the presence of publication bias was not a problem in the current study because it would take a relatively large number of statistically non-significant unpublished results to change the significant effect of fermented cassava on FI, FCR, and ADG in broiler chickens**.**

## Conclusion

The pooled results revealed that dietary fermented cassava increased feed intake and feed conversion ratio while reducing average daily gain in broilers. Conversely, subgroup analysis showed that Hubbard broiler strain fed cassava fermented with a blend of *A. charticola* and *Bacillus subtilis* at inclusion levels of 1–10 % and 11–20 % for 1 to 42 days had improved growth performance. Pooled results indicated that dietary fermented cassava reduced carcass yield, but did not affect cut-part and visceral organ weights. Results from the present meta-analysis indicate that the incorporation of fermented cassava to the diets of broiler chickens increased serum cholesterol, but had no effect on abdominal fat content, triglyceride, high-density lipoprotein, and low-density lipoprotein. In contrast, fermented cassava increased the concentrations of white blood cells, total protein, creatinine, and uric acid in broiler chickens, but had no influence on red blood cell, haemoglobin concentration, and packed cell volume with evidence of significant heterogeneity. However, studied modifiers explained most of the sources of heterogeneity in the present meta-analysis. It is therefore recommended that more research be conducted to determine factors responsible for the poor feeding value of fermented cassava in broiler chickens as to maximise its potential as an energy source in the poultry industry.

## Data Availability

Data will be made available on reasonable request.
